# Effectiveness of Therapeutic Exercise and Patient Education on Cancer-Related Fatigue in Breast Cancer Survivors: A Randomised, Single-Blind, Controlled Trial with a 6-Month Follow-Up

**DOI:** 10.3390/jcm11010269

**Published:** 2022-01-05

**Authors:** Virginia Prieto-Gómez, María José Yuste-Sánchez, Javier Bailón-Cerezo, Helena Romay-Barrero, Irene de la Rosa-Díaz, Cristina Lirio-Romero, María Torres-Lacomba

**Affiliations:** 1 Physiotherapy in Women’s Health (FPSM) Research Group, Physiotherapy Department, Faculty of Medicine and Health Sciences, University of Alcalá, 28805 Madrid, Spain; v.prieto@uah.es (V.P.-G.); marijo.yuste@uah.es (M.J.Y.-S.); bailonfisioterapia@gmail.com (J.B.-C.); irenedelarosadiaz@gmail.com (I.d.l.R.-D.); Cristina.Lirio@uclm.es (C.L.-R.); 2 Department of Nursery, Physiotherapy and Occupational Therapy, Faculty of Physiotherapy and Nursery, University of Castilla-La Mancha, 45071 Toledo, Spain; Helena.Romay@uclm.es; 3 Centro Superior de Estudios Universitarios La Salle, Department of Physical Therapy, Universidad Autónoma de Madrid, 28049 Madrid, Spain

**Keywords:** cancer-related fatigue, persistent pain, breast cancer, therapeutic exercise, patient education

## Abstract

This study aimed to determine the effectiveness of therapeutic exercise plus patient therapeutic education on perceived fatigue, functional capacity and pain in breast cancer survivors with cancer-related fatigue. A randomised, single-blind, clinical trial was conducted with a total of 80 breast cancer survivors who presented cancer-related fatigue. Women were randomised into a supervised therapeutic exercise group (STE-G) (*n* = 40) or an unsupervised exercise group (UE-G) (*n* = 40). Both interventions included patient therapeutic education and were delivered in three sessions per week over eight weeks. The main outcome was perceived fatigue as assessed by the Spanish version of the Functional Assessment of Chronic Illness Therapy-Fatigue subscale (FACIT-F). Other evaluated outcomes were pain measured on a visual analogue scale, and distance measured using the 6-Minute Walk Test. Data were collected at baseline, immediately post-intervention, and at three and six months after baseline. Significantly greater improvements across all variables were observed in the STE-G throughout the entire follow-up period with the exception of pain. Conclusions: A supervised therapeutic exercise program plus patient therapeutic education significantly reduce perceived fatigue and increase functional capacity in breast cancer survivors suffering from cancer-related fatigue compared to an unsupervised physical exercise program based on individual preferences with patient therapeutic education.

## 1. Introduction

Cancer and its treatments are usually accompanied by symptoms that affect body function and health-related quality of life, such as fatigue, pain, depression, or insomnia [1,2]. Several studies have shown associations between elevated plasma levels of pro-inflammatory cytokines [i.e., tumour necrosis factor Alpha (TNFα) and interleukin 6 (IL6)] and increased levels of fatigue, pain, sleep disturbance, and depressive symptoms in cancer survivors, which may underly the etiopathogenesis [1].

Cancer-related fatigue (CRF) is one of the side effects associated with poorer health-related quality of life outcomes in cancer survivors [3,4] and entails a negative impact on socioeconomic status, greater healthcare utilisation, and reduced survival rates [2,5]. Complex multifactorial processes have been associated with the development of CRF, including physiological (adaptive responses to inflammation, dysregulation of the hypothalamic-pituitary-adrenal axis, or reduced energy metabolism), clinical (direct or secondary effects of cancer treatments and contributions from comorbidities), and psychological (depression, catastrophising, or fear of recurrence) components [2]. CRF can be experienced across all stages of the disease (active treatment, maintenance, and survivorship or disease-free) [6]. Up to 85% of patients under active treatment suffer from CRF, with 9–45% being moderate to severe [2]. Severe fatigue is found in 20–40% of breast cancer (BC) survivors [7], of which around 30% report significant CRF ten years after treatment [8]. Despite the high prevalence, CRF is seldom addressed in therapeutic plans for cancer survivors [9].

Several pharmacologic and non-pharmacologic interventions have been described to treat CRF [2]. While various drugs have been investigated, the evidence supports combined therapeutic exercise (TE) and psychological interventions as the best therapeutic option for CRF management [5], showing both efficacy and safety for reducing fatigue in BC survivors [10]. Recent systematic reviews [10,11,12,13] support the benefits of aerobic and resistance exercise in patients with CRF in agreement with the Guidelines for Exercise Testing and Prescription in the cancer population by the American College of Sports Medicine (ACSM) [14,15]. In addition, TE is recommended in several clinical practice guidelines to reduce other adverse effects derived from cancer treatments, such as pain, depression, or bone and body weigh alterations [10]. Nevertheless, randomised clinical trials with higher methodological quality and longer follow-up periods are needed to determine the optimal exercise parameters and dosage [9,10,11,12,13,14]. Several studies have stated the superior effect of supervised versus unsupervised modalities of exercise [12,13], largely due to the different exercise intensity and adherence. However, the proposed control interventions (stretching, maintenance of usual physical activity levels, or low-intensity physical activity) do not encourage regular physical exercise (150–300 min/week of moderate or 75–150 min/week of vigorous aerobic exercise) despite having been previously associated with a lower risk of postmenopausal BC [16] nor take into account the individual preferences of women, which could promote adherence to the TE programs [12,13]. Furthermore, adherence to TE is a key factor in the efficacy of the treatment [17], similarly to other physical therapy interventions. Since only a small proportion of cancer survivors think that CRF can be effectively managed [18], the combination of TE interventions with patient therapeutic education, as recommended for chronic pain conditions, could increase adherence and improve fatigue and related symptoms [19].

However, the evidence supporting the efficacy of supervised versus unsupervised exercise under equal conditions of adherence is insufficient. Therefore, a randomised clinical trial was conducted to test the hypothesis that, under similar conditions of adherence to exercise, supervised TE combined with a therapeutic education program could be more effective in reducing fatigue and pain and improving functional capacity in BC survivors with CRF compared to unsupervised exercise of their preference plus an educational strategy.

## 2. Materials and Methods

### 2.1. Design

A single-centre, single-blind, 2-arm parallel randomised clinical trial was conducted in BC survivors with CRF at the Physiotherapy Unit for Women’s Health Research of the University of Alcalá (Madrid, Spain) between July of 2018 and October of 2020. The study was registered at ClinicalTrials.gov (NCT02828189) and approved by the Research Ethics Committee of the University of Alcalá (CEI2013011). Participant consent was obtained in all cases, and all procedures were performed in accordance with the Declaration of Helsinki and the CONSORT statement.

### 2.2. Participants

Women who were diagnosed with CRF were recruited in a consecutive order by the Physiotherapy in Women’s Health Research Group at the University of Alcalá (Madrid, Spain). The inclusion criteria were: cancer-free women who had undergone unilateral BC surgery with chemotherapy or radiation therapy (RT); having completed their adjuvant RT and/or chemotherapy treatment at least 6 months before the beginning of the trial; diagnosis of CRF by their family doctor according to ICD-10 [20] criteria; and having suffered from CRF for at least 6 months, with or without persistent pain over the same period. The criteria for exclusion were: women diagnosed with bilateral BC; suffering from acute pain; movement alterations or cardiorespiratory disability contraindicating exercise; medication intake, such as pain relievers and/or non-steroidal anti-inflammatories and/or bronchodilators; neurologic conditions or cognitive limitations preventing the understanding of information for participation in the study or treatment instructions; over 85 years or younger than 18 years of age.

### 2.3. Sample Size Estimation

Statistical power and sample size were estimated to detect a between-group difference of 1.4 points in perceived fatigue as scored on the Functional Assessment of Chronic Illness Therapy-Fatigue (FACIT-F) subscale with a 5-point variance. This *a priori* estimation was based on the findings of a previous pilot study carried out *ad hoc* to test the methods and estimate the sample size. We estimated a sample size of 40 individuals in each arm for a power of 80%, an alpha level of 0.05, and a potential maximum dropout rate of 10%. The statistical software Granmo 7.12 (Institut Municipal d’Investigació Mèdica, Barcelona, Spain, 2012) was used for sample size calculation.

### 2.4. Randomisation and Blinding

After checking compliance with the inclusion criteria and reading the study information, the eligible women signed the informed consent form for participation. Participants were individually assessed before the intervention(V0) (MJYS: Pt1) and randomly allocated (*n* = 40 in each group) into a supervised therapeutic exercise group (STE-G) or an unsupervised exercise group (UE-G) by an independent physical therapist (MTL: Pt2) blind to the group assignment. The randomisation was performed using a computer randomisation list at a ratio of 1:1 (EPIDAT v.3.1, Xunta Galicia, Spain). Following baseline assessment, Pt2 disclosed the group allocation to the two physiotherapists delivering the interventions (VPG: Pt3 & IRD: Pt4) as well as to the participants via a phone call.

### 2.5. Assessment and Data Collection

Three follow-up visits were scheduled: immediately after completing the intervention (V1), and at three (V2) and six months (V3) after baseline. These follow-up appointments were arranged to match participant availability, who were also contacted by phone or text message one week and three days in advance to confirm or reschedule the date.

A different physiotherapist specialised in women’s health (Pt1), who remained blind to participant group allocation throughout the trial, performed all assessments (V0–V3). The baseline assessment was conducted on the day the women agreed to participate in the study and prior to randomisation, and subjects were instructed not to reveal their allocation to Pt1 to ensure blinding success.

Personal and clinical data collected at V0 included age, education level, employment status, BC surgery, postoperative complications, adjuvant therapies, pain intensity, duration of pain, and CRF symptoms. Pt2 recorded the primary and secondary outcomes at V0 and follow-up assessments.

#### 2.5.1. Primary Outcome

Perceived fatigue was assessed with the Spanish version of the FACIT-F scale [21], a self-reported unidimensional scale to evaluate perceived fatigue and its impact on daily activities. It comprises 13 items that are rated on a 5-point Likert-type scale so that the total score ranges from 0 (worst outcome) to 52 (best outcome). The FACIT-F is easy to administer, reliable, valid, and responsive to change [21,22]. A minimum clinically important difference (MCID) of 3 points is required to be clinically significant in the BC population [23].

#### 2.5.2. Secondary Outcomes

Secondary outcomes were pain reported on a visual analogue scale (VAS) and distance in metres using the 6-Minute Walk Test (6MWT).

The participating women reported their pain intensity over the last two days [24] on a 100-mm VAS consisting of a 100-mm horizontal line marked with “no pain” on the left end and “worst imaginable pain” on the right end. Previous research [25] has reported pain intensity measured with the VAS to be reproducible and valid. A MCID of 9–11 mm is required to reach clinical significance in the BC population [26].

The 6MWT is a simple and safe test used to objectively assess functional capacity. Women were asked to walk as far as possible along a 30-m minimally-trafficked corridor for six minutes, and the traveled distance in metres was recorded as the outcome measure. The 6MWT is a valid and reliable instrument tested in cancer patients [27]. A MCID of 25 m is required to be clinically significant [28].

### 2.6. Interventions

The two interventions lasted 8 weeks, with three weekly sessions of 50–60 min each except for the first 6 weeks that included therapeutic education, so the duration was longer by 30 min in two of the three weekly sessions in the experimental group or by 50 additional weekly minutes in the control group. Therapeutic education was imparted at the Physiotherapy in Women’s Health Research Unit of the University of Alcalá (Madrid, Spain). The patient therapeutic education for BC survivors suffering from fatigue and persistent pain contained specific content on healthy lifestyle habits and pelvic floor health (patient therapeutic education description can be found elsewhere) [29,30]. Patient therapeutic education was taught to all participants in small groups (6 women maximum). Two physiotherapists (Pt2 and Pt3) with more than ten-year experience in physiotherapeutic management of BC patients delivered the interventions in the STE-G and UE-G, respectively. The physiotherapists Pt1, Pt2, and Pt3 were the only research team members aware of participant group allocation.

The exercise program for the STE-G followed ACSM’s Guidelines for Exercise Testing and Prescription in the cancer population [14,15,31,32] and was conducted in small groups (6 women maximum) in an equipped space of the Physiotherapy in Women’s Health Research Unit of the University of Alcalá (Madrid, Spain). The participants wore heart rate monitors during all sessions. The designed sessions included: (1) 5 min of warm-up (mobility and ludic aerobic exercises); (2) 30 min of moderate-to-vigorous intensity aerobic exercise using a step at 65 to 85 % of the maximum heart rate [14,15]; 15 min of progressive resistance training with elastic bands for major muscle groups, performed in one to three series of 8–12 repetitions with a one-minute resting time, at moderate intensity reported as a perceived exertion of 3–4 [31]; and finally, 10 min of stretching exercises for the major muscle groups exercised during the sessions. Moreover, breathing exercises were performed for the recovery of thoracic and diaphragmatic flexibility and breathing re-education to achieve diaphragmatic ventilation [33,34].

Patients in the UE-G were instructed to perform 50–60 min of autonomous physical exercise at home based on individual preferences three times a week. Individual preferences for activities included progressive march on flat ground (*n* = 16, 40%), dancing (*n* = 10, 25%), or cycling (*n* = 14, 35%). The program consisted of: (1) 5 min of warm-up (mobility and ludic aerobic exercises); (2) 30–45 min of their preferred exercise at moderate intensity reported as a perceived exertion of 3–4 [31], and (3) 10 min of stretching exercises of the major muscle groups exercised during the sessions.

Adherence to exercise was monitored during the intervention and follow-up by means of a one-week diary [one A4 page (×24); three items per week; type of exercise (open); frequency (tick), duration (number), comments (open)]. The exercise diaries were completed by the patients from both groups and collected weekly during the first six weeks of the intervention, immediately after the intervention (week 8), and at each follow-up assessment (weeks 12 and 24).

### 2.7. Data Analysis

Descriptive statistics were used for the intergroup comparison of participant characteristics, relevant clinical variables, perceived fatigue, self-reported pain, and distance in metres during the 6MWT at baseline. Separate linear regression models were generated to estimate the average change in continuous outcomes (FACIT-F, 6MWD, and pain VAS) since the baseline at subsequent assessments (V1, V2, and V3), adjusting for the basal value. As basal values are frequent covariates, removing the variance associated to such covariates (adjustment) avoids a potential bias resulting from imbalances in baseline values between participants. The Student’s *t*-test for independent samples was used for the adjusted means in the comparison between groups. The results of the intergroup comparisons are represented by their adjusted mean with the relevant confidence interval at 95% (CI95%) and corresponding *p*-value.

A repeated-measures generalised linear model was employed for the assessment of all outcomes in the intragroup comparison, where the repeated measures (assessment visits) were the intrasubject factor and the intervention group was the intersubject factor. A full factor model was used and the type III sum of squares was estimated. Homogeneity between assessments was evaluated using Mauchly’s sphericity test, yielding statistical significance for all measured outcomes (*p*-value < 0.001). Thus, the Greenhouse-Geisser correction was used to analyse the presence of changes throughout the different visits and whether this evolution was similar between groups. These results are presented in graphs, which include the *p*-values of the Greenhouse-Geisser comparisons. An α = 0.05 was set for all tests. All statistical tests were performed using Stata Data Analysis and Statistical Software (Version 10, StataCorp LP, College Station, TX, USA).

The therapeutic adherence for each group was calculated as a percentage by dividing the average TE sessions that women performed over the 24-week program by the total number of TE sessions in the program (72 sessions).

## 3. Results

**Figure 1:**
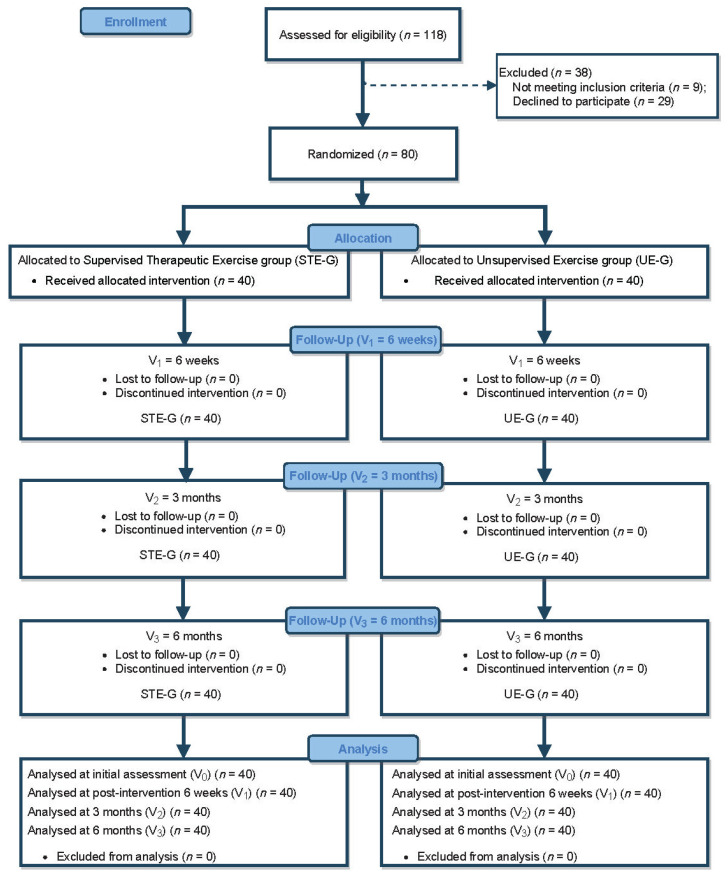
Flow diagram of participants throughout the trial.

A total of 80 BC survivors met the inclusion criteria and were randomly allocated into two groups (STE, *n* = 40; UE, *n* = 40) with no dropouts or losses to follow-up (see flow diagram in Figure 1). No intergroup differences were observed at baseline (Table 1).

### 3.1. Primary Outcome

Significant intergroup differences were found in the FACIT-F scores (*p* < 0.001) and MCID at all post-treatment visits (V1, V2, and V3).

### 3.2. Secondary Outcomes

No significant intergroup differences (*p* > 0.05) in the pain VAS were found at any of the three post-intervention assessments (V1, V2, and V3). In contrast, significant intergroup differences in the 6MWT travelled distance (*p* < 0.001) and MCID were observed at all follow-up visits (V1, V2, and V3).

Table 2 shows the average changes since the baseline for each outcome variable and intervention group with their corresponding 95%CI and *p*-values.

**Figure 2:**
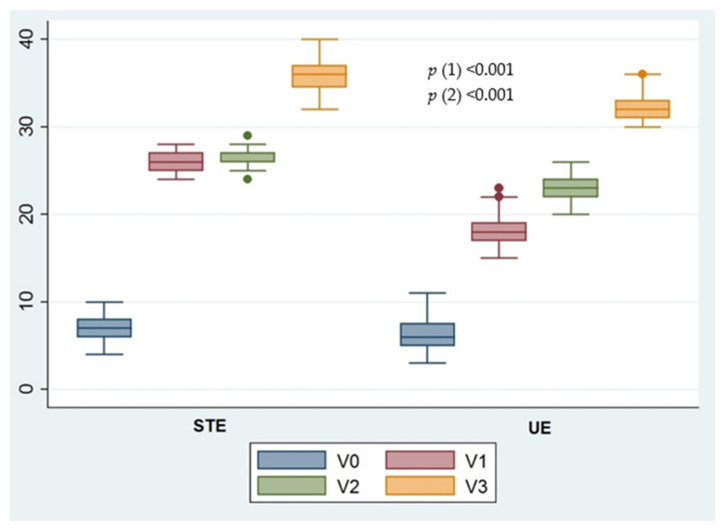
Evolution of the main outcome (fatigue) since the baseline for each intervention group. STE: Supervised therapeutic exercise; UE: Unsupervised exercise. *p*-value (1), Greenhouse-Geisser correction to contrast the mean change throughout the assessment visits. *p*-value (2), Greenhouse-Geisser test to verify whether the evolution of the FACIT-F score is similar in the STE and UE groups.

Figure 2 shows the evolution of the perceived fatigue and health-related quality of life outcomes since the baseline for each intervention group.

No adverse events or medical conditions such as lymphoedema, unbearable pain, or pelvic floor dysfunctions were recorded.

Adherence to exercise during the intervention and the follow-up period was similar in both groups (STE-G: 95%; UE-G: 87.5%). The women in this study showed high therapeutic adherence to the TE programmes.

## 4. Discussion

The present study evaluated the effectiveness of two interventions consisting of supervised or unsupervised TE in combination with patient education on perceived fatigue, functional capacity, and pain after the intervention and at three and six months of follow-up in BC survivors suffering from CRF.

A greater improvement in perceived fatigue and functional capacity was observed in BC survivors who underwent STE plus patient therapeutic education versus those who performed UE combined with patient therapeutic education.

The present clinical trial was designed according to the standardised method for reporting exercise programs in clinical trials [35]. Therefore, the current study presents two methodological strengths: a 6-month follow-up period and the inclusion of a therapeutic education program combined with an exercise program based on participant preferences to promote adherence to treatment [36,37].

Perception of fatigue as measured by the FACIT-F scale was very low at baseline in both groups compared to the general population [38,39]. Although the score considerably increased in both groups until reaching moderate levels at V3, statistically significant and clinically relevant differences in favour of the STE-G were found throughout all assessments. Similarly, the women in both groups showed reduced cardiorespiratory fitness at V0 resulting in a decreased effort capacity as measured by the distance walked in the 6MWT [40]. This may be because the most prevalent comorbidity in this population is cardiovascular disease [41]. The travelled distance during the 6MWT was significantly lower in BC survivors compared to healthy women [40] and was lower than in the reference trial in the case of the present study [40]. The peak aerobic capacity (VO_2_peak) during cardiorespiratory exercises is widely considered as the gold standard for measuring cardiovascular capacity, which directly correlates with fatigue levels, functional capacity, and health-related quality of life [27]. However, the covered distance in the 6MWT was chosen in the present study as an indirect measure of cardiorespiratory capacity that allows to estimate both cardiorespiratory fitness [27,42] and data related to functional capacity [28], for which the 6MWT is considered a bedside tool requiring less human and material resources and that is closer to the usual clinical context.

Although lung function and alterations in breathing capacity were not specifically assessed in the present study, the decrease in lung function following BC treatments is considered as a possible conditioning factor for the appearance and/or perpetuation of fatigue. Thus, the STE program included an approach for the recovery of thoracic and diaphragmatic flexibility, as well as for re-educating breathing to achieve diaphragmatic ventilation. This approach aimed to mitigate some side effects of adjuvant-therapy, such as the decreased lung function in the medium and long term caused by RT. Side effects of RT such as thickening of the pleura, tissue contractions, atelectatic areas, or elevation of the ipsilateral hemidiaphragm can be found in 87% of BC women [43,44]. Nevertheless, prior or concomitant chemotherapy treatment with RT increases the degree of pulmonary toxicity, similarly to combined hormonal therapy and RT. The incidence of pulmonary fibrosis in women treated with the hormonal therapy drug tamoxifen is higher compared to that in patients treated with aromatase inhibitors [45]. In addition, RT and surgery can also lead to rib cage impairment, affecting the mobility of the upper limb due to the damage caused to the skin, fasciae, and muscles that decreases rib cage mobility and contributes to the decrease of pulmonary function [43,46]. This study aimed to achieve a significant improvement in the perception of fatigue in the women included in the STE-G versus those in the UE-G by incorporating this approach as a contributing factor for the amelioration of internal and external ventilatory mechanics [33,34].

In addition, several studies have reported the superiority of supervised TE over other unsupervised TE modalities. Supervision enhances therapeutic adherence, promotes individualisation and safety of the exercise program, and reinforces trust between the health professional and patient [12,13]. Although all participants received a therapeutic education program that promoted adherence and which was equally monitored in both groups, the direct supervision of exercises probably improved this parameter [12]. Also, the combination of aerobic and resistance exercise [11,14] likely contributed to the high adherence observed, since both moderate-to-vigorous TE intensity and the combination of both modalities of physical exercise have been shown to be a key factor in improving CRF.

Persistent pain is a common problem among women being treated for BC, with a prevalence following treatment ranging from 24% to 47% [47]. The causes underlying persistent pain are multifactorial and are directly related to the physical sequels derived from surgery and adjuvant treatment, in addition to other physical and psychosocial risk factors [47,48]. At the baseline, the intensity of pain perceived by the women in both groups was moderate [49] (mean values of 5.60 cm and 5.55 cm on the VAS in the STE-G and UE-G, respectively). These values are in agreement with the findings of a previous study by Juhl et al. in a sample of 100 women with persistent pain, of which 50% reported moderate pain values (4 to 7 points on a numerical rating scale) [47]. In terms of the effect on pain relief, pain intensity decreased in both groups after an 8-week TE program. This is in agreement with the findings by Sandal et al. that showed important reductions in pain following 8 to 12 weeks of exercise therapy. Nevertheless, a single session of exercise can result in exercise-induced hypoalgesia [50], a phenomenon that may occur by means of aerobic [51] or isometric exercise [52]. The educational intervention, which included tools for the self-management of perceived pain, is another factor that may have contributed to the clinically relevant reduction in pain intensity observed in both groups, which was already evident at V1 and progressively improved until V3. Therapeutic education plays a fundamental role in the development and self-management of chronic pathologies, is associated with improvements in health-related quality of life, and enhances adherence to the guidelines and recommendations of health professionals [29,30]. In this sense, the high adherence to therapeutic education observed in this study (95% in the STE-G and 87.5% in the EU-G) could be related to the therapeutic education program delivered to all the women. This program specifically focused on encouraging the communication between the physiotherapist and patients, autonomy, and the acquisition of abilities affecting perceived self-efficacy [29,30,37]. Furthermore, no participant was lost to follow-up. SMS reminders were sent one week and three days before each follow-up visit and the appointment date or time could be changed upon patient request. This flexibility is part of the strategies for improving adherence to follow-up appointments and likely contributed to the lack of dropouts in the present study [53].

Among the main strengths of this study is the fact that the resources required for assessing the measured variables and implementing the program are easily transferable to routine clinical practice, thus facilitating the inclusion of effective, safe, and easy-to-administer TE in programs for women with BCF. Furthermore, none of the women included in this study developed lymphoedema secondary to BC, not even those in the STE-G who performed specific strength-resistance exercises. These results reinforce the evidence that supervised, individualised, and progressive TE of the upper limb does not increase the likelihood of developing or worsening lymphoedema in BC survivors suffering from, or at risk of, developing lymphoedema [54,55,56,57]. TE also has shown a positive direct effect on functional capacity and health-related quality of life. Moreover, to the authors’ knowledge, this is the first study to integrate pelvic floor care [29] into exercise programmes for BC survivors. Of note, 58% of BC survivors report urinary disorders (stress and urge incontinence, dysuria, and urinary tract infections) [58] and up to 78% suffer from sexual dysfunctions (42% dyspareunia) [59]. This may be because hormone therapy aggravates the symptoms of genitourinary syndrome of menopause, mainly vaginal dryness and dyspareunia. Up to 70% of affected women show concern for their pelvic and vaginal health as well as their sexual function [60]. Furthermore, age, obesity, menopause, and exercise [61,62] are exacerbating factors of these pelvic floor dysfunctions, so any exercise program directed to these women should adapt to their situation and integrate pelvic health components.

One of the main limitations of this study was the difficulty of comparing the results to previous studies on the effectiveness of TE for CRF management due to heterogeneity in participant characteristics, stage of the oncologic process, associated comorbidities, diagnostic instruments, and characteristics of the intervention (type of exercise, exercise dosage, frequency, intensity, etc.) in these studies. In addition, no device or application was available to corroborate if appropriate levels of exercise intensity were reached in the medium- and long-term, nor were results of pelvic floor dysfunctions collected, despite having incorporated pelvic floor health into the program. Finally, controlling drugs intake, such as pain relievers, was not possible.

## 5. Conclusions

A supervised TE program combined with patient therapeutic education significantly reduces perceived fatigue and increases functional capacity compared to an autonomous physical exercise program based on individual preferences combined with patient education in BC survivors suffering from CRF. However, even though pain intensity progressively decreased in all participants, no significant differences were observed between groups. Future studies are needed that include the assessment of lung function and integrate individualised respiratory physiotherapy prior to and/or concomitant with exercise interventions.

## Figures and Tables

**Table 1:** Intergroup comparison at baseline. Values are expressed as numbers with percentages in parenthesis unless stated otherwise.

Characteristics	STE Group (*n* = 40)	UE Group (*n* = 40)	Total Sample (*n* = 80)	*p*-Value
Age years, Median (IQI)	53.5 (58.8–47.0)	54.5 (63.0–48.5)	54.0 (62.0–47.3)	0.846
Education level, frequency (%)				
Basic	1 (2.5)	0 (0)	1 (1.3)	0.626
Primary education	13 (32.5)	11 (27.5)	24 (30.0)	
Secondary education	16 (40.0)	16 (40.0)	32 (40.0)	
Pre-university	5 (12.5)	9 (22.5)	14 (17.5)	
University or HNUE	5 (12.5)	4 (10.0)	9 (11.3)	
Currently employed, frequency (%)	16 (40.0)	18 (45.0)	34 (42.5)	0.821
Surgical procedure, frequency (%)				
Mastectomy	0 (0)	1 (2.5)	1 (1.3)	0.542
Quadrantectomy	11 (27.5)	5 (12.5)	16 (20)	
Tumorectomy	29 (72.5)	34 (85)	63 (78.8)	
Axillary dissection procedure frequency (%)				
ALND	38 (95)	40 (100)	78 (97.5)	0.473
SLNB	2 (5)	0 (0)	2 (2.5)	0.472
Postoperative complications frequency (%)				
Seroma	0 (0)	1 (2.5)	1 (1.3)	0.999
SLT	37 (92.5)	33 (82.5)	70 (87.5)	0.310
Lymphedema	4 (10)	2 (5)	6 (7.5)	0.671
Postoperative therapy frequency (%)				
Radiotherapy	40 (100)	40 (100)	80 (100)	
Chemotherapy	38 (95)	40 (100)	78 (97.5)	0.473
Hormonal therapy	32 (80)	26 (65)	58 (72.5)	0.210
Time (months) since treatment, Median (IQI)	9 (5)	7.5 (3.75)	8 (5)	0.110
VAS (mm), $\bar{X}$ (SD)	5.60 (1.6)	5.55 (1.5)	5.57 (1.5)	0.885
6MWD (m), $\bar{X}$ (SD)	349.50 (114.1)	304.5 (113.2)	327 (115.1)	0.080
FACIT-F, $\bar{X}$ (SD)	7.03 (1.7)	6.33 (1.9)	6.63 (1.8)	0.086

STE: Supervised therapeutic exercise; UE: Unsupervised exercise; IQI: Interquartile interval; $\bar{X}$: mean; SD: Standard deviation; HNUE: Higher non-university education; ALND: Axillary lymph node dissection; SLNB: Sentinel lymph node biopsy; SLT: Superficial lymphatic thrombosis; VAS: Visual analogue scale; 6MWD: Six-minute walking distance; FACIT-F: Functional Assessment of Chronic Illness Therapy-Fatigue scale.

**Table 2:** Average changes in outcome variables since the baseline (adjusted by basal values).

		Supervised Therapeutic Exercise Group		Unsupervised Exercise Group		Supervised Therapeutic Exercise Group vs. Unsupervised Exercise Group
Outcome		Mean Difference	95%CI	Mean Difference	95%CI	*p*-Value
VAS (mm)	V_1_	−2.05	−2.58 to −1.51	−1.62	−2.16 to −1.08	0.258
VAS (mm)	V_2_	−2.50	−3.04 to −1.95	−2.73	−3.23 to −2.21	0.543
VAS (mm)	V_3_	−3.08	−3.54 to −2.60	−3.72	−3.72 to −2.72	0.661
FACIT-F	V_1_	19.17	18.68 to 19.67	11.92	11.10 to 12.74	<0.001
FACIT-F	V_2_	19.70	19.20 to 20.19	17.10	16.33 to 17.86	<0.001
FACIT-F	V_3_	28.77	27.94 to 29.60	26.10	25.17 to 27.02	<0.001
6MWD (m)	V_1_	207.37	190.37 to 224.37	132.37	119.03 to 145.72	<0.001
6MWD (m)	V_2_	288.00	265.10 to 310.89	186.37	163.23 to 209.51	<0.001
6MWD (m)	V_3_	306.75	275.35 to 338.14	200.25	172.69 to 227.31	<0.001

V: Visit (assessment); VAS: Visual analogue scale; 6MWD: Six-minute walking distance; FACIT-F: Functional Assessment of Chronic Illness Therapy-Fatigue scale; NA: Not applicable.

## Data Availability

Data are held securely by the research team and may be available upon reasonable request and with relevant approvals in place.
